# Liquid Biopsy and Potential Liquid Biopsy-Based Biomarkers in Philadelphia-Negative Classical Myeloproliferative Neoplasms: A Systematic Review

**DOI:** 10.3390/life11070677

**Published:** 2021-07-10

**Authors:** Mihnea-Alexandru Găman, Matei-Alexandru Cozma, Elena-Codruța Dobrică, Sanda Maria Crețoiu, Amelia Maria Găman, Camelia Cristina Diaconu

**Affiliations:** 1 Faculty of Medicine, “Carol Davila” University of Medicine and Pharmacy, 050474 Bucharest, Romania; drcameliadiaconu@gmail.com; 2 Department of Hematology, Center of Hematology and Bone Marrow Transplantation, Fundeni Clinical Institute, 022328 Bucharest, Romania; 3 Department of Gastroenterology, Colentina Clinical Hospital, 020125 Bucharest, Romania; matei.cozma@gmail.com; 4 Department of Pathophysiology, University of Medicine and Pharmacy of Craiova, 200349 Craiova, Romania; codrutadobrica@gmail.com (E.-C.D.); gamanamelia@yahoo.com (A.M.G.); 5 Department of Dermatology, “Elias” University Emergency Hospital, 011461 Bucharest, Romania; 6 Department of Cell and Molecular Biology and Histology, “Carol Davila” University of Medicine and Pharmacy, 050474 Bucharest, Romania; 7 Clinic of Hematology, Filantropia City Hospital, 200143 Craiova, Romania; 8 Department of Internal Medicine, Clinical Emergency Hospital of Bucharest, 105402 Bucharest, Romania

**Keywords:** myeloproliferative neoplasms, polycythemia vera, essential thrombocythemia, primary myelofibrosis, liquid biopsy, extracellular vesicles, microvesicles, microparticles, circulating endothelial cells, cell-free DNA

## Abstract

Myeloproliferative neoplasms (MPNs) are rare, clonal disorders of the hematopoietic stem cell in which an uncontrolled proliferation of terminally differentiated myeloid cells is noted. Polycythemia vera (PV), essential thrombocythemia (ET) and primary myelofibrosis (PMF) are included in the category of Philadelphia-negative, so-called classical MPNs. The potential applications of liquid biopsy and liquid biopsy-based biomarkers have not been explored in MPNs until now. Thus, a systematic search was computed in PubMed/MEDLINE, Web of Science and The Cochrane Library and, in total, 198 potentially relevant papers were detected. Following the removal of duplicates (*n* = 85), 113 records were screened. After the exclusion of irrelevant manuscripts based on the screening of their titles and abstracts (*n* = 81), we examined the full texts of 33 manuscripts. Finally, after we applied the exclusion and inclusion criteria, 27 original articles were included in this review. Overall, the data analyzed in this review point out that liquid biopsy and liquid biopsy-based biomarkers (cell-free DNA, extracellular vesicles, microparticles, circulating endothelial cells) could be used in MPNs for diagnostic and prognostic purposes. Future research is needed to clarify whether this technique can be employed to differentiate between MPN subtypes and secondary causes of erythrocytosis, thrombocytosis and myelofibrosis, as well as to predict the development of thrombosis.

## 1. Introduction

Myeloproliferative neoplasms (MPNs) are rare, clonal disorders of the hematopoietic stem cell in which an uncontrolled proliferation of terminally differentiated myeloid cells is noted. Polycythemia vera (PV), essential thrombocythemia (ET) and primary myelofibrosis (PMF) are included in the category of Philadelphia-negative, so-called classical MPNs, as opposed to chronic myeloid leukemia in which the Philadelphia chromosome, i.e., a shorter chromosome 22 resulting from a reciprocal translocation between the long arms of chromosomes 9 and 22, respectively, can be identified [1,2,3]. MPNs are known to exhibit phenotypic mimicry, can transform into secondary myelofibrosis or evolve to acute leukemia, and are marked by thrombotic complications which can often be the cause of death in these hematological disorders [4,5,6,7]. Thrombosis is often the inaugural event in the diagnosis of MPNs and should alarm the clinician to screen the patient for MPNs particularly if it occurs in the territory of cerebral or splanchnic veins which are considered unusual sites for such complications. MPNs subjects can experience both arterial and venous thrombosis, with thrombotic events being viewed as major culprits in the morbidity and mortality of this patient subgroup [5].

Due to advancements in the fields of precision hemato-oncology and personalized medicine, the diagnosis, monitoring and management of blood cancers have allowed for the employment of less invasive techniques to understand disease biology and to identify new prognostic markers. Consequently, the concepts of liquid biopsy and liquid biopsy-based biomarkers, i.e., the detection and use of circulating tumor fragments from body fluids rather than solid cancer tissue, in hematological disorders have attracted much interest, and have been successfully applied in the management of leukemias or lymphomas [8,9,10,11].

Despite the fact that liquid biopsy generally refers to the detection of cell-free DNA (cfDNA), in blood cancers the concept has been extended by some authors to include not only gene-based, but also cell-based or protein-based biomarkers, such microparticles (MPs), circulating endothelial cells (CECs) or extracellular vesicles (EVs) [9,11]. Elevated cfDNA, CECs or EVs have been detected in lymphoma, acute myeloid leukemia, acute lymphoblastic leukemia and chronic lymphocytic leukemia. In acute myeloid leukemia, the concentration of CECs has been viewed as an indicator of the metabolic activity of the disease, as it seems to have been associated with the response to chemotherapy and with the prognosis. Similarly, CECs have been regarded an indicator of disease aggressiveness in chronic lymphocytic leukemia. Moreover, in acute myeloid leukemia, high exosome (a type of EVs) levels in comparison with healthy counterparts have been reported [11]. However, the potential applications of liquid biopsy or liquid biopsy-based biomarkers have not been explored in MPNs until now. Although the examination of the bone marrow via tissue biopsy remains a major diagnostic criterion for ET and PMF (particularly to differentiate between prefibrotic PMF and ET) and a minor diagnostic criteria for PV and that MPNs can be monitored using peripheral blood samples, the discovery of new biomarkers to inform prognosis and thrombotic risk, as well as to help elucidate the phenotypic mimicry of MPNs and enable a better characterization of disease biology, are warranted [1,3,4,5]. 

Thus, the objective of our systematic review was to summarize the latest findings regarding the potential role that liquid biopsy and liquid biopsy-based biomarkers could play in the diagnosis, monitoring and management of MPNs. 

## 2. Materials and Methods

The current systematic review was prepared based on The Preferred Reporting Items for Systematic reviews and Meta-Analyses (PRISMA) protocol [12].

Three investigators (M.-A.G., M.-A.C. and E.-C.D.) conducted an independent literature search in PubMed/MEDLINE, Web of Science and The Cochrane Library for manuscripts published from the inception of these databases up to 7 May 2021. The keywords and word combinations used are depicted in the Supplementary Materials (Tables S1–S3).

The studies were included in this systematic review if they met the following inclusion criteria: 1. Original articles or research letters evaluated the potential role of liquid biopsy in the diagnosis, management, evolution or prognosis of patients diagnosed with classical MPNs (PV, ET or MF) OR evaluated the potential role in the diagnosis, management, evolution or prognosis of patients suffering from classical MPNs of biomarkers/molecules that could be detected using a liquid biopsy from peripheral blood samples, i.e., circulating tumor cells, circulating tumor clusters, exosomes, extracellular vesicles, microparticles or circulating endothelial cells. 2. The subjects recruited in these original studies were adults (aged ≥ 18 years) and had a certified MPN diagnosis; 3. The papers were published in English, French, Italian or Romanian (the languages spoken by the authors); 4. The manuscripts provided sufficient data regarding the following data of interest: study location, study design, type of MPN, number of MPN patients recruited, methods employed to assess the biomarkers that could be detected using a liquid biopsy, results regarding the detection of liquid biopsy-based biomarkers or the results of the liquid biopsy; 5. The full-text of the identified manuscripts could be downloaded or retrieved. We selected the following exclusion criteria: 1. Reviews, case reports, meeting abstracts, grey literature; 2. Papers depicting non-clinical studies (research conducted in vitro or on animals). 3. Studies conducted in children (age < 18 years); 3. The papers were published in languages unknown to the authors (e.g., Chinese); 4. The papers did not report sufficient data on the outcomes of interest; 5. The full text of the articles was unavailable to the authors.

We evaluated study eligibility by screening the titles and abstracts of the potentially relevant papers, followed by the examination of the full texts of eligible manuscripts. The aforementioned process was conducted independently by three investigators (M.-A.G., M.-A.C. and E.-C.D.) who also computed a customized Microsoft Excel spreadsheet by extracting the following data from the eligible studies: first author surname, publication year, study location, study design, type of MPN, number of MPN patients recruited, methods employed to assess the biomarkers that could be detected using a liquid biopsy, main results of the analyzed papers. Any disagreement was resolved with the help of the senior authors (S.M.C., A.M.G. and C.C.D.).

The methodological quality of the studies was assessed using the methodological index for non-randomized observational studies (MINORS) [13]. The risk of bias of the analyzed studies was evaluated using the Mixed Methods Appraisal Tool (MMAT) [14].

## 3. Results

**Figure 1:**
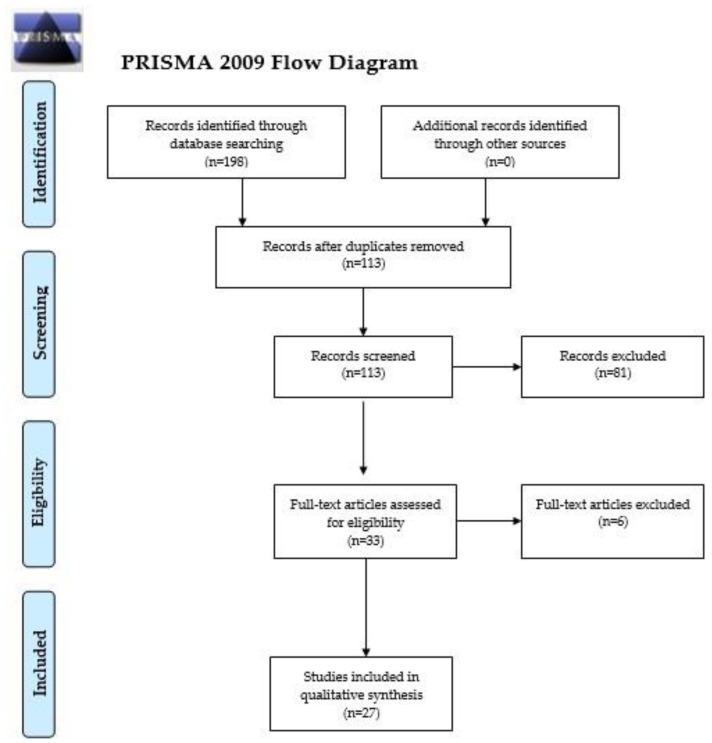
PRISMA 2009 Flow Diagram. From Moher, D.; Liberati, A.; Tetzlaff, J.; Altman, D.G. The Prisma Group (2009). Preferred reporting items for systematic reviews and meta-analyses: The PRISMA statement. PLoS Med 6(7): e1000097, doi:10.1371/journal.pmed.1000097. For more information, visit www.prisma-statement.org. Accessed on 9 May 2021 [12].

In total, 198 potentially relevant papers were detected. Following the removal of duplicates (*n* = 85), 113 records were screened. A flowchart diagram of the detailed steps of the literature search process is depicted in Figure 1. After we excluded the irrelevant manuscripts based on the screening of their titles and abstracts (*n* = 81), we examined the full texts of 33 manuscripts. Finally, after we applied the exclusion and inclusion criteria, 27 original articles were included in this review [15,16,17,18,19,20,21,22,23,24,25,26,27,28,29,30,31,32,33,34,35,36,37,38,39,40,41].

Overall, the 27 studies assessed included 1336 patients diagnosed with classical MPNs and were conducted predominantly in Europe (*n* = 22; *n* = 10 for Italy, *n* = 4 for France), followed by Asia (*n* = 4) and Australia (*n* = 1). Most studies were observational (cross-sectional: *n* = 25) in design and included all subtypes of classical MPNs (*n* = 8), whereas others analyzed ET in particular (*n* = 7), PV in particular (*n* = 3) or PV+ET (*n* = 3). The analyzed studies investigated the levels of cell-free DNA (*n* = 1), extracellular vesicles (*n* = 7), microparticles (*n* = 14) or circulating endothelial cells (*n* = 5) levels in MPNs. Flow cytometry was by far the most employed method of detection (*n* = 23) [15,16,17,18,19,20,21,22,23,24,25,26,27,28,29,30,31,32,33,34,35,36,37,38,39,40,41]. 

### 3.1. Cell-Free DNA

Only one study assessed cell-free DNA (cfDNA) levels in classical MPNs [15]. 

The quantity of cfDNA in plasma samples collected from MPN subjects was assessed by Garcia-Gisbert et al. (2020), who discovered that there is an elevated amount of cfDNA in the plasma of MPNs, irrespective of the subtype (PV, ET or PMF), versus healthy counterparts (*p* < 0.001 for all three subtypes). In addition, as compared to PV and ET subjects, PMF patients displayed elevated cfDNA (*p* < 0.001 for both comparisons), a finding which remained statistically significant (*p* < 0.001 for both comparisons) even when the cfDNA/leukocyte ratio was calculated. The authors also pointed out the positive cfDNA-leukocyte count and cfDNA-LDH associations (r = +0.220, *p* = 0.023 and r = +0.532, *p* < 0.001, respectively), but there was no correlation of cfDNA and thrombocytes, hematocrit or hemoglobin values. The positive cfDNA-LDH association was stronger in PMF (r = 0.679, *p* = 0.008), but it was statistically significant when the PV and ET subjects were viewed as a collective entity (r = +0.442, *p* < 0.001). MPN subjects who were diagnosed following a thrombosis or who experienced such an event during follow-up had elevated cfDNA (*p* = 0.038) versus MPNs without a history of thrombosis. This finding was valid only when the MPNs were analyzed collectively, as statistical significance was not reached when PV, ET and PMF were evaluated separately. Moreover, the researchers evaluated the mutational profile of MPN patients using next-generation sequencing in both DNA samples extracted from granulocytes and cfDNA and evidenced that cfDNA was as accurate as granulocyte DNA for the detection of driver (*JAK2*, *CALR*, *MPL*) and non-driver (*TET2*, *ASXL1*, *IDH2*, *DNMT3A*, *SF3B1*, *SRSF2*, etc.) mutations. In addition, the variant allele frequency of the mutations was lower in granulocytes versus cfDNA (*p* < 0.001), although these parameters displayed a positive association (r = +0.897, *p* < 0.001). The variant allele frequency of *JAK2* (*p* < 0.001, in particular for ET), *MPL* (*p* = 0.003) and *SRSF2* (*p* = 0.043), but not of *CALR* or other non-driver mutations, was elevated in cfDNA versus granulocyte DNA. Interestingly, the amount of cfDNA was positively associated with the number of mutations per subject (r = +0.242, *p* = 0.012), particularly in PMF (r = +0.572, *p* = 0.033) and was elevated in patients harboring mutations of the *MPL* or *ASXL1* genes versus non-mutated cases (*p* = 0.034 and *p* = 0.019). The variant allele frequency of *JAK2* V617F did not change in cfDNA nor granulocyte DNA in a subject with PV who was managed with hydroxyurea, yet interferon employment in ET reduced this parameter in both cfDNA and granulocyte DNA samples [15].

### 3.2. Extracellular Vesicles

A total of seven studies investigated the levels of extracellular vesicles (EVs) in MPNs by the analysis of the following parameters: megakaryocyte-derived EVs (MK-EVs), platelet-derived EVs (PLT-EVs), microvesicles (MVs), megakaryocyte-derived MVs (MK-MVs), platelet-derived MVs (PLT-MVs), monocyte-derived MVs (MMVs) and endothelial-derived MVs (EMVs) [16,17,18,19,20,21,22].

In a pilot study, Barone et al. (2019) assessed the potential role of MVs as biomarkers of MPNs in 81 subjects (MF, *n* = 61; ET, *n* = 20). Patients with MF (*p* < 0.001 and *p* < 0.01, respectively) and ET (*p* < 0.001 and *p* < 0.001, respectively) had significant lower levels of MKMVs and higher levels of PLTMVs compared to controls. Moreover, in MF, platelet-MVs were detected in lower concentrations as compared to ET (*p* < 0.01). Subjects with PMF and SMF had similar levels of MVs. MF patients who harbored mutations of the *JAK2* or *CALR* genes displayed an elevated number of PLTMVs versus patients diagnosed with triple-negative MPNs (*p* < 0.05 for both) and controls (*p* < 0.001 and *p* < 0.05, respectively). The presence of *JAK2* (*p* < 0.001) or *CALR* (*p* < 0.01) mutations or the absence of any mutation (*p* < 0.01) in MF was also linked with reduced levels of MKMVs versus healthy comparators. Triple-negative MF subjects had lower concentrations of PLTMVs versus *CALR*(+) or *JAK2* V617F(+) cases. The presence of *JAK2* (*p* < 0.05 and *p* < 0.001, respectively) or *CALR* mutations (*p* < 0.05 and *p* < 0.01, respectively) was linked with reduced MKMVs and increased concentrations of PLTMVs in ET versus controls and in ET versus triple-negative subjects, respectively. ET subjects displayed similar levels of MVs irrespective of their genetic status. In MF subjects with high/intermediate-2 risk as calculated by the International Prognostic Scoring System (IPSS), elevated PLTMVs and lower MKMVs concentrations were registered versus intermediate-1/low-risk counterparts and healthy subjects (*p* < 0.05, *p* < 0.01 and *p* < 0.001, respectively), particularly if they harbored *JAK2* or *CALR* mutations and suffered from IPSS high-risk MF (*p* < 0.001). The researchers demonstrated a positive association between of the concentrations of MKMVs with the number of thrombocytes in the peripheral blood (r = +0.45, *p* < 0.001), as well as a negative association with the degree of splenomegaly (r = −0.39, *p* < 0.01) in MF. In addition, the authors detected a negative association of MKMVs and IL-6 (r = −0.38, *p* < 0.05), and a positive association of *p*-selectin (r = +0.36, *p* < 0.01) and of thrombopoietin (r = +0.51, *p* < 0.01) with PLTMVs in MF. Furthermore, Barone et al. (2019) explored the impact of ruxolitinib treatment on MVs in MF. The subjects who displayed a reduction in the degree of splenomegaly had lower PLTMVs and elevated MKMVs (*p* < 0.001) at baseline. The authors also argued, based on the study of the receiver-operator characteristic curve, that subjects with MKMVs < 19.95% would fail respond to ruxolitinib treatment. At 6 months after ruxolitinib initiation, an increase in MKMVs (*p* < 0.001) and a reduction in PLTMVs (*p* < 0.01) was exhibited by the subjects in whom ruxolitinib treatment was successful. EMVs and MMVs were also elevated in MF (*p* < 0.01 and *p* < 0.05, respectively). Ruxolitinib reduced the concentrations of EMVs only in the patients who displayed a decrease of the degree of splenomegaly following ruxolitinib initiation, although at baseline all MF patients had similar EMVs and MMVs. MMVs remained unaltered in MF patients following ruxolitinib treatment [16].

Barone et al. (2020) investigated how cytokines (CK), namely several interleukins (IL-1β, IL-6 and IL-10) and tumor necrosis factor-α (TNF-α), were expressed on EVs originating in the monocytes (MEVs) of MF subjects and the impact of ruxolitinib treatment on these molecules. MEVs-CK were similar between MF subjects and controls in normal conditions. However, following lipopolysaccharide stimulation, the researchers detected an elevation (*p* < 0.001) in the MEVs-CK of controls but not of MF subjects. Surprisingly, following treatment with ruxolitinib, the levels of MEVs-CK were elevated in MF. Ruxolitinib also altered the expression of IL-1β, IL-10 and TNF-α receptors on the circulating monocytes of MF patients [17].

Poisson et al. (2020) isolated MVs from the blood of subjects diagnosed with *JAK2* V617F-positive MPNs subjects and studied the impact of these MVs on the endothelial wall of mice aortas. The presence of plasma-derived MVs collected from MPNs subjects enhanced the phenylephrine-induced contraction in the aorta of mice in comparison with MVs-free MPNs plasma or the plasma of control subjects (*p* < 0.01). However, the researchers did not clearly mention from which MPNs subtypes the samples were collected from [18]. Pecci et al. (2015) assessed the presence of particulate cytoplasmic structures in samples of peripheral blood collected from five MPNs subjects. In MPNs, these elements were detected only in thrombocytes and granulocytes, being absent in RBCs, monocytes and lymphocytes. Similarly, proteasome concentrations were also elevated in the protein extracts of thrombocytes and granulocytes of MPN patients versus healthy counterparts (*p* < 0.001) [19]. Caivano et al. (2015) evaluated the characteristics of microvesicles (MVs) in hematological disorders, including in patients with PMF (*n* = 5). The investigators detected that, in PMF, there are elevated concentrations of small-diameter (0–0.3 μm) MVs (*p* = 0.008) [20].

Fel et al. (2019) aimed to characterize the proteome of EVs-rich serum collected from subjects diagnosed with PV. Transferrin receptor protein 1 and heparanase were 13 times (*p* < 0.001) and 11.2 times (*p* < 0.001) higher in the EVs of PV subjects versus controls. Moreover, plasminogen activator inhibitor 1 (*p* = 0.003), histone H4 (*p* = 0.001), angiogenin (*p* < 0.001) and histone H2B (*p* = 0.008) were detected in amounts 5 to 6 times higher versus controls. The concentrations of matrix metalloproteinase-9, neurogenic locus notch homolog (Notch) protein 3, lysozyme C, histone H3, L-selectin, lactotransferrin and solute carrier family 2 were 4–5 times higher in PV, whereas coagulation factor XI, myeloperoxidase, C-reactive protein, vinculin and platelet multimerin-1 were 3–4 times higher. Overall, PV was associated with elevated number of cells and activated thrombocytes, as well as inflammatory, immune, angiogenic and procoagulant markers. PV was characterized by an enhanced number of CD42d+, CD71+ and CD62L+ cells, thus the EVs might be derived from PLTs, RBCs and (or) monocytes, respectively [21].

Forte et al. (2021) investigated the characteristics of circulating CD34+ cells in the bloodstream of subjects suffering from *JAK2* V617F-positive or triple-negative MF in comparison to healthy controls. The number of CD34+ cells in the circulation was nearly 6 times more elevated in triple-negative versus *JAK2* V617F-positive MF (*p* = 0.01), in addition to an elevated co-expression of CD63, CD133 and CD184 (*p* = 0.04, *p* = 0.03 and *p* = 0.03, respectively). However, the in vitro survival rate and the clonogenic capacity were similar. In terms of signaling pathways, *IL6-JAK-STAT3* (*p* < 0.01) and *KRAS* (*p* < 0.01) signatures were enhanced in *JAK2* V617F-positive and triple-negative MF, respectively. Moreover, in the triple-negative as compared to *JAK2* V617F-positive cases, a downregulation of genes related to inflammation (*NLRP3*, *IL9-R*, *CD48*, *CD180*, *MMP2* and *ROBO1*) occurred. In the CD34+ cells sampled from triple-negative MF patients, the researchers detected an upregulation of several genes linked to cellular adhesion (*GP5*, *PCDHB9*, *PCDHB11*, *PCDHB12* and *PCDHB14*) and of several genes that worked to inhibit apoptosis (*FCMR*, *TNFRSF1B*, *TSPYL5* and *PTPN13*) was present. In addition, the same cells displayed downregulated pro-apoptotic (*TP53INP1* and *TNFSF10*) and adhesion-linked (*AMICA1*, *CDCP1*, *CNTNAP2*, *ITGAL*, *LGALS3BP*, *MMP2*, *MLLT4*, *SERPINB8* and *ROBO1*) genes. Pro-inflammatory cytokines were also detected in elevated levels in triple-negative MF subjects (*p* < 0.05 for IL-12 and IL-1β) versus controls and *JAK2* V617F-positive subjects (*p* < 0.01 for IL-1β, TPO and IFN-γ; *p* < 0.05 for TNF-α; *p* < 0.001 for IL-12) versus controls. In *JAK2* V617F-positive versus triple-negative MF, higher concentrations of TPO were registered (*p* < 0.05), particularly in women (*p* = 0.02). In addition, the authors sought to characterize EVs isolated from MF subjects and demonstrated that *JAK2* V617F-positive and triple-negative MF patients had EV similar in size and morphology, and all depicted a low positivity for CD81, CD63 and CD9. However, EVs originating in megakaryocytes were detected in low values versus controls in both MF genetic subtypes. In terms of EVs originating in thrombocytes, their levels were reduced healthy controls and triple-negative MF subjects versus *JAK2* V617F-positive patients (*p* < 0.05). The EVs sampled from triple-negative MF subjects presented some peculiarities, however, namely enhanced in vitro survival (*p* < 0.05) and overexpression of miRNAs, in particular miR-361-5p (*p* = 0.04). As compared to controls, in *JAK2* V617F-positive and triple-negative MF there was an upregulation of miR-34a-5p (*p* = 0.005 and *p* = 0.02), miR-222-3p (*p* = 0.05 for both), miR-361-5p (*p* = 0.05 for both) and miR-127-3p (*p* = 0.03 only in *JAK2* V617F-positive MF). The authors also noted a positive association of miR-34a-5p (r = +0.738, *p* = 0.02) with the variant allele frequency of *JAK2* V617F, as well as a negative association of miR-212-3p (r = −0.72, *p* = 0.03) with the same parameter [22].

### 3.3. Microparticles

A total of 14 studies focused on the role of MPs originated from platelets (PMPs), red blood cells (RMPs), granulocytes (GMPs), monocytes (MMPs), the endothelium (EMPs) or on MPs expressing the tissue factor (TF+MPs), platelet microaggregates (PMAs), platelet-monocyte conjugates (PMCs), platelet-neutrophil conjugates (PNCs) or activated platelets (APs) [23,24,25,26,27,28,29,30,31,32,33,34,35,36].

Zhang et al. (2017) aimed to evaluate the impact of circulating MPs in 92 patients diagnosed with MPNs and observed that, in classical MPNs, EMPs (*p* < 0.01 for PMF and ET, *p* < 0.05 for PV), RMPs (*p* < 0.01 for PMF, *p* < 0.05 for ET and PV), PMPs (*p* < 0.01 for PMF, *p* < 0.05 for ET and PV) and TF+MPs (*p* < 0.01 for PMF, *p* < 0.05 for ET and PV) were detected in elevated concentrations as compared to healthy controls. Moreover, patients diagnosed with PMF were reported to have higher circulating levels of RMPs (*p* < 0.05), PMPs (*p* < 0.05), EMPs (*p* < 0.05) and TF+MPs (*p* < 0.05) versus patients suffering from PV. In addition, ET subjects had higher levels of EMPs versus PV subjects (*p* < 0.05). The presence of splenomegaly and a positive history for thrombotic complications were associated with elevated concentrations of RMPs, PMPs, EMPs and TF+MPs (*p* < 0.05 for all) in the MPN group. In terms of genetics, the presence of the *JAK2* V617F mutation was linked with higher levels of PMPs (*p* < 0.05) as opposed to MPN cases who did not harbor this mutation [23].

Villmow et al. (2003) assessed the levels of PMPs, PMAs, APs, PNCs and PMCs in 47 patients with *BCR-ABL1-negative* MPNs (*n* = 37) and CML (*n* = 10) versus healthy controls. APs, expressed as the percentage of CD62p-positive cells, were higher in both *BCR-ABL1-positive* and *BCR-ABL1-negative* MPNs (*p* < 0.05 for all) versus healthy counterparts. The highest median percentage was registered in MF (14.5%), followed by CML (13.8%), PV (12.0%) and ET (11.5%) versus controls (9.00%). However, PMPs were only elevated (*p* < 0.05) in *BCR-ABL1-negative* MPNs (12% in PV, 11.0% in ET and MF), with lower median percentages noted in CML (6.0%) and healthy subjects (5.2%). In this study, irrespective of the subtype of MPN, PMAs concentrations were similar to control subjects. PNCs were only significantly elevated in ET (10.3%) and PV (8.3%) versus healthy counterparts (6.8%), whereas the authors reported similar PNCs values to controls in MF and CML. The study group also depicted higher percentages of PMCs versus PNCs. PMCs were the highest among PV (15.4%) and ET (15.0%), percentages which were significantly higher versus controls (*p* < 0.05). PMCs were similar between CML (12.7%), MF (10.9%) and controls (8.0%). Although most thrombotic complications occurred in PV (77%) and ET (58%) versus MF (42%) and CML (10%), patients with a positive history for thrombotic or hemorrhagic events or those who received blood-thinning medication had similar percentages of APs, PMPs, PMAs, PNCs and PMCs when compared to the MPN subjects who experienced no such complications (*p* > 0.05 for all) [24].

Trappenburg et al. (2009) evaluated the levels of plasma-derived MP in 21 patients diagnosed with ET and detected higher levels of MPs in ET versus controls (*p* = 0.039). The authors argued that phosphatidylserine is encompassed in the cell membrane of the detected MPs as these molecules bonded to annexin-V. Moreover, since >95% of MPs were CD41(+) and (or) CD61(+), the researchers demonstrated that the largest amount of the identified MPs originated from thrombocytes and were, thus, PMPs. Moreover, CD61(+) PMPs were detected in significantly elevated numbers in ET subjects versus healthy counterparts (*p* = 0.043). However, controls had higher CD63(+) PMPs versus ET patients (*p* < 0.001), with CD63 being a marker of APs. In addition, ET patients displayed higher numbers of EMPs (27% of MPs in ET patients versus 1% in controls), i.e., CD62E(+) (*p* = 0.007) and CD144(+) (*p* = 0.021), as well as GMPs, i.e., CD66b(+) and CD66acde(+), or MMPs, i.e., CD14(+). Interestingly, 90% of EMPs also coexpressed CD41, accounting for nearly a quarter of MPs that expressed CD41. The authors observed that the EMPs which were positive for both CD62E and CD41 were more likely to be detected in ET patients harboring at least one cardiovascular risk factor (*p* = 0.045). Among the MPs, TMPs, i.e., CD4(+)/CD8(+), BMPs, i.e., CD20(+), ICAM(+), i.e., CD54(+), and VCAM(+), i.e., CD106(+) cells were represented in small quantities (<1%) and were similar between ET subjects and controls. The presence of the *JAK2* V617F mutation or the treatment regimens of the subjects had no impact of the origin or quantity of any MPs, including TF+MPs [25].

Taniguchi et al. (2017) analyzed the levels of circulating MPs in 59 subjects diagnosed with MPNs, but failed to detect a significant difference between the concentrations of MPs in MPNs versus controls, including in terms of MPs that displayed annexin V or TF positivity. Moreover, MPs levels were similar between MPN subtypes as well. Although the employment of cytoreductive therapy did not influence the total concentrations of MPs in MPNs, it did decrease the levels of procoagulant, annexin V(+) MPs (*p* < 0.05) and TF+MPs (*p* < 0.05), but there was no difference between the effects on MPs of hydroxyurea and anagrelide, nor did they cause any phenotypic changes of these MPs. Anticoagulants, namely warfarin, also seemed to decrease MPs in MPNs (*p* = 0.02). Furthermore, the authors assessed the phenotype of the MPs and discovered that nearly 70% of the annexin V(+) MPs were in fact PMPs, i.e., CD41a(+), whereas the remaining 30% were either EMPs, i.e., CD146(+), or CD45(+), i.e., leukocyte-derived. The percent of CD146(+)/CD45(+), i.e., lymphocyte-derived MPs was negligible. Although the source of TF+MPs in the control group was discovered to be the endothelium, the researchers failed to discover the cellular origin of approximately half of the TF+MPs in MPNs. Moreover, the relationship between procoagulant MPs in MPNs and the production of thrombin remained ambiguous. There was no association of procoagulant MPs and the complete blood count or any parameters that assessed the hemostasis or fibrinolysis of MPNs subjects. Interestingly, the presence of the *JAK2* V617F mutation did not exert a statistically significant impact of the concentrations of TF+MPs or annexin V(+) MPs. However, a previous history of thrombotic events (*p* = 0.02), particularly in subjects who were not managed with cytoreductive therapy (*p* = 0.01), was linked with elevated levels of TF+MPs. In addition, in the univariate but not in the multivariate analysis, more than 84.7 TF+MPs per microliter and the presence of documented CV risk (not expressed by the presence of the *JAK2* V617F mutation, sex or age >60 years) predicted the development of thrombosis in MPN subjects (*p* = 0.02 for both) [26].

Tan et al. (2013) investigated the role of MPs in 23 subjects with PV, revealing a higher count of MPs in PV versus secondary polycythemia or controls (*p* < 0.001 for both). In PV, the detected MPs showed positivity for lactadherin and were classified into PMPs, RMPs, GMPs and EMPs. PMPs and RMPs accounted for the biggest share (approximately 85% and 7.5%, respectively) of MPs in PV. Although the total MPs, PMPs, RMPs, GMPs and EMPs counts were elevated in PV versus secondary polycythemia or healthy subjects (*p* < 0.001 for all), TF+MPs (which displayed positivity for CD142) concentrations were similar in PV, secondary polycythemia and controls. Interestingly, there was no association of complete blood count parameters and MPs levels in PV, but the thrombocytes and red blood cells of PV patients the exposure of phosphatidylserine on the membrane of these cells was elevated versus secondary polycythemia (*p* < 0.01 for both) or controls (*p* < 0.001 for both). Moreover, the MPs, red blood cells and thrombocytes of PV subjects were responsible for a hypercoagulable state in this disorder, as expressed by a reduction in clotting time (*p* < 0.01) and an elevated production of thrombin and intrisinc FXase complex (*p* < 0.01). PV patients managed with hydroxyurea displayed a reduction in total circulating MPs (*p* = 0.01), RMPs (*p* = 0.008), PMPs (*p* = 0.03), and the exposure of phosphatidylserine on the membrane of red blood cells (*p* = 0.04) and thrombocytes (*p* = 0.01). Cytoreduction did not alter EMPs and GMPs concentrations (*p* > 0.05 for both) [27].

Piccin et al. (2017) analyzed the levels of circulating MPs in ET patients, reporting that in ET subjects on risk-adapted therapy, particularly acetylsalicylic acid alone or in combination with hydroxyurea, total MPs and PMPs, i.e., CD61(+) and CD36(+), were lower versus controls or anagrelide-treated ET patients (*p* < 0.001). Moreover, RMPs, which displayed CD235 positivity, and EMPs (positive for CD62E) were detected in larger amounts (*p* < 0.001 for both) in ET patients who did not receive treatment versus treated patients and controls. Furthermore, controls had lower concentrations of EMPs (positive for CD105) versus ET subjects, with the lowest EMPs levels registered in ET patients who were managed with anagrelide and acetylsalicylic acid (*p* = 0.015). TF+MPs, i.e., CD142(+), were measured in similar concentrations in all study groups. Interestingly, ET patients who were prescribed the combination of hydroxyurea and acetylsalicylic acid displayed elevated levels of nitric oxide and adrenomedullin (*p* < 0.001) versus the other subgroups. The employment of the combination of anagrelide and acetylsalicylic acid in ET was linked with reduced endothelin-1 concentrations [28].

Ahadon et al. (2020) evaluated MPs levels in 15 patients with PV versus 15 healthy control subjects. Flow cytometry was employed to detect MPs that displayed annexin V, CD61 and CD144 positivity. PMPs were detected in elevated numbers in PV patients versus controls (1.3% versus 0.65%, *p* < 0.01). EMPs were; however, similar between the two subgroups (*p* = 0.43). Despite the increased number of thrombocytes and PMPs in the PV group, the researchers registered no linear correlation between the two aforementioned parameters [29].

Aswad et al. (2020) used flow cytometry to quantify PMPs and RMPs concentrations, as well as their activity (using functional assays) in a group of 179 patients with MPN (99 with ET, 51 with PV, 29 with PMF) and 20 healthy patients. In MPNs, the number of PMPs was greater than the number of RMPs and both were significantly higher in MPNs versus controls (*p* < 0.001). The pro-coagulation activity of MPs was also elevated in MPNs versus controls (*p* < 0.001). The pro-coagulation activity of PMPs positively associated with PMPs levels (*p* = 0.029). However, in MPN subjects with a positive history for thrombotic events, lower PMPs values (*p* < 0.001) were detected. The presence of the *JAK2* V617F mutation was linked with higher PMPs concentrations versus *JAK2* V617F-negative cases (*p* = 0.029). There was no relationship of RMPs and thrombotic events or the *JAK2* V617F mutation. RMPs were registered in similar levels between MPN subtypes. However, in PV and ET, as opposed to PMF, PMPs concentrations were elevated (*p* = 0.008 and *p* = 0.014, respectively). In addition, in MPNs, PMPs levels were associated with several parameters of the complete blood count, e.g., hemoglobin and hematocrit (*p* < 0.001 for both), PLTs (*p* = 0.002), RBCs (*p* = 0.027) and leukocytes (*p* = 0.023) [30].

Charpentier et al. (2016) evaluated the relationship between MPs, the risk of thrombosis and the mutational status in 74 ET subjects. Patients harboring mutations of the *JAK2* gene displayed elevated concentrations of total MPs (*p* < 0.001), RMPs (*p* < 0.01), PMPs (including PMPs with P-selectin co-expression, *p* < 0.001 for both), but similar levels of MMPs, GMPs and EMPs in comparison with ET patients harboring mutations of the *CALR* gene or triple-negative ET cases. Triple-negative and *CALR*-mutated ET shared similar concentrations of MPs, irrespective of the measured subtype. In terms of the impact of MPs on coagulation in ET, there was an association of MPs with thrombin generation (r = +0.47, *p* = 0.01) and with the phospholipid-dependent pro-coagulant activity of the MPs (r = +0.53, *p* = 0.0002). In particular, patients harboring mutations in the *JAK2* gene, as opposed to the *CALR* gene or triple-negative ET cases, displayed MPs with enhanced pro-coagulant activities (*p* = 0.01). In addition, ET patients at high risk of thrombotic events as calculated by the IPSET (International Prognostic Score of thrombosis in World Health Organization-essential thrombocythemia) score exhibited increased concentrations of MPs versus intermediate/low-risk cases (*p* < 0.001). In particular, the authors identified a predictor of high thrombotic risk in ET patients, i.e., >4600 circulating MPs per microliter [31].

Marchetti et al. (2014) explored the role of circulating MPs in the ET-linked status of hypercoagulation in 73 subjects diagnosed with ET versus 72 counterparts with a normal status of health. In terms of thrombin generation, the lag-time (*p* < 0.05) and time to peak (*p* < 0.05) were reduced in ET subjects versus controls, whereas the endogenous thrombin potential (*p* < 0.05) and peak of thrombin (*p* < 0.05) were higher in controls as compared to ET patients. Patients harboring the *JAK2* V617F mutation registered elevated values for endogenous thrombin potential (*p* < 0.05) and peak of thrombin (*p* < 0.05) and lower values for lag-time (*p* < 0.05) and time to peak (*p* < 0.05) when compared to their *JAK2* V617F-negative counterparts. In addition, the clotting times of the subjects suffering from ET were significantly shorter (*p* < 0.001) versus controls, particularly in *JAK2* V617F-positive patients (*p* < 0.05 versus *JAK2* V617F-negative cases). The *JAK2* V617F mutation emerged as a predictor of shorter clotting times in ET patients (*p* = 0.012, β = −0.311, B = −8.46). The plasma phospholipid procoagulant activity of plasma samples collected from ET patients was positively associated with the lag-time (r = +0.323, *p* < 0.001) and time to peak (r = +0.335, *p* < 0.0001), as well as negatively associated with the peak of thrombin (r = −0.406, *p* < 0.0001) and endogenous thrombin potential (r = −0.353, *p* < 0.0001). When the MPs were removed from the collected samples, the clotting times increased, whereas the endogenous thrombin potential decreased, in both controls (*p* < 0.001) and ET subjects (*p* < 0.001). The circulating levels of tissue factor were elevated in ET patients versus controls (*p* < 0.001), irrespective of the *JAK2* mutational status. The FVIIa/AT complex concentrations in the plasma were also elevated in ET versus controls (*p* < 0.01), but the *JAK2* V617F mutation seems to play a key role in this finding, as only *JAK2* V617F-positive cases seemed to display higher levels of FVIIa/AT complex in the plasma versus controls (*p* < 0.05) [32].

Moles-Moreau et al. (2009) investigated the diagnostic utility of PMPs, reticulated thrombocytes and CD36 expression in subjects with ET versus subjects with reactive thrombocytosis and healthy controls but discovered that these parameters cannot be employed to discriminate ET from reactive thrombocytosis. However, ET patients had higher values of CD36+ cells (*p* < 0.001), PMPs/total number of thrombocytes (*p* = 0.009), reticulated thrombocytes (*p* = 0.006) and absolute reticulated thrombocytes (*p* = 0.09), but a similar total number of PMPs (*p* = 0.0221), versus subjects exhibiting reactive thrombocytosis. As compared to healthy controls, ET patients had higher values of CD36+ cells (*p* < 0.001), PMPs (*p* < 0.001), PMPs/total number of thrombocytes (*p* < 0.001), reticulated thrombocytes (*p* < 0.001) and absolute reticulated thrombocytes (*p* < 0.001). Patients suffering from reactive thrombocytosis had similar values of CD36+ cells (*p* = 0.44) and reticulate thrombocytes (*p* = 0.10) versus their healthy counterparts but PMPs (*p* < 0.001), PMPs/total number of thrombocytes (*p* < 0.001) and absolute reticulated thrombocytes (*p* < 0.001) counts were elevated [33].

Connor et al. (2013) evaluated the reactivity of thrombocytes and PMPs (displaying positivity for annexin V and CD41) concentrations in patients with malignant (myelodysplastic syndromes and ET) and non-malignant (immune thrombocytopenia) hematological disorders. ET subjects were predominantly females, had elevated thrombocyte counts and elevated levels of PMPs (*p* = 0.0041) versus controls. Cells from ET subjects had an increased expression of CD62p but not of CD63 and an increased PAC-1 binding in unstimulated whole blood, whereas CD63 exposure decreased following collagen-related peptide and thrombin receptor activating peptide stimulation and CD62p exposure decreased following stimulation with collagen-related peptide. No changes in CD62p or PAC-1 occurred following stimulation with thrombin receptor activating peptide or adenosine diphosphate [34].

Duchemin et al. (2010) assessed the circulating procoagulant activity of plasma collected from MPN subjects, demonstrating that this parameter was elevated (particularly in *JAK2* V617F-positive cases with a homozygous genotype), whereas endogenous thrombin potential was reduced, in MPNs versus healthy subjects (*p* < 0.001 and *p* = 0.004, respectively). However, after the filtration of MPs from MPNs-collected samples, endogenous thrombin potential remained lower and the circulating procoagulant activity remained high versus in controls (*p* = 0.017 and *p* < 0.001, respectively). The authors evidenced that MPNs are linked with MPs-related resistance to thrombomodulin and decreased concentrations of free protein S but not protein C or factor VIII (*p* = 0.001) versus controls. Moreover, positive associations of neutrophil (r = 0.416, *p* = 0.001 and r = +0.333, *p* = 0.01), RBCs (r = +0.371, *p* = 0.004 and r = +0.446, *p* < 0.001) and PLTs (r = +0.327, *p* = 0.01 and r = +0.272, *p* = 0.04) values, but no association with a positive history for thrombosis, and the circulating procoagulant activity in MPNs. Patients receiving treatment (*p* = 0.006), particularly cytoreduction-based regimens (*p* = 0.018), displayed lower circulating procoagulant activity. The endogenous thrombin potential was also associated with the *JAK2* V617F allele burden (r = −0.283, *p* = 0.031), whereas following the filtration of MPs, MPN subjects with *JAK2* V617F homozygous genotype displayed the lowest values of this parameter, as well as the highest circulating procoagulant activity [35].

Kissova et al. (2015) repeatedly measured the circulating levels of MPs in subjects suffering from *BCR-ABL1*-negative MPNs and investigated their impact on the occurrence of thrombotic events in these patients. As opposed to healthy controls who had MPs concentrations in the normal range, in MPNs elevated MPs concentrations were constantly detected (*p* < 0.001). Moreover, when the procoagulant activity of these MPs was analyzed, the authors reported that it increased particularly in PV versus ET or PMF (88% versus 73.2% and 68.3%, respectively; *p* = 0.002), as well as in *JAK2* V617F-positive (78.1%) versus *JAK2* V617F-negative (66.7%) MPNs (*p* = 0.007), particularly in those subjects with an elevated allele burden (*p* = 0.001). Furthermore, in PV subjects, it was associated with hemoglobin and hematocrit values (r = +0.323 and r = +0.275, respectively; *p* = 0.002 and *p* = 0.008, respectively). In addition, it was also increased (*p* = 0.029) in MPN subjects with a positive history for venous thrombotic events (84.7%) versus those who had not experienced episodes of venous thrombosis (73.2%). The use of cytoreductive agents was linked to a decreased MPs procoagulant activity (72.8 % versus 85.5%, *p* = 0.010). In addition, the number of thrombocytes, but not of leukocytes or neutrophils, were associated with an elevated MPs procoagulant activity (*p* = 0.006) [36].

### 3.4. Circulating Endothelial Cells

A total of five studies assessed circulating endothelial cells (CECs) in MPNs [37,38,39,40,41].

Alonci et al. (2008) evaluated the levels of CECs (progenitor cells expressing CD34) in MPNs, revealing elevated CD34+ CECs levels in PMF (*p* = 0.007), ET (*p* = 0.04) and PV (*p* = 0.04) versus controls. PV (*p* < 0.001) and ET (*p* < 0.01) subjects displayed lower CD34+ CECs values as compared to PMF. CECs co-expressing CD34+, CD133+ and VEGFR2 were elevated in PMF (*p* < 0.01) and PV (*p* < 0.01) but similar to controls in ET (*p* > 0.05). CECs co-expressing CD34+ and VEGFR2, but negative for CD133, were elevated in PMF (*p* < 0.01; *p* < 0.05 versus PV), PV (*p* = 0.002) and ET (*p* = 0.002) versus controls [37].

Belotti et al. (2011) measured CECs (positive for CD146, negative for CD45) concentrations in ET versus healthy subjects and detected lower CECs concentrations in the latter (*p* < 0.0001). The presence of the *JAK2* V617F mutation, a positive history for thrombosis or the employment of cytoreduction versus antiplatelet therapy did not impact on CECs levels in ET, nor were CECs levels associated with age, sex, ET duration or complete blood count parameters. The concentrations of soluble E-selectin were also elevated in ET, irrespective of the *JAK2* V617F mutational status, as compared to controls (*p* = 0.0369) [38].

Torres et al. (2013) assessed circulating levels of progenitor and non-progenitor CECs in patients with PV, ET and venous thromboembolism in comparison with healthy subjects. CECs levels were elevated in both MPN subjects (*p* < 0.001 for the entire group, *p* = 0.001 for PV, *p* = 0.001 for ET) and patients who had experienced a venous thrombosis (*p* < 0.001). However, progenitor CECs were detected in lower amounts in patients who had experienced a venous thrombosis (*p* = 0.029 versus controls), but similar between the healthy subjects and MPNs (irrespective whether PV or ET). In terms of the quantity of CECs that expressed markers of adhesion and procoagulation, only CD62E+ CECs were elevated in MPNs, irrespective of subtype (*p* < 0.001 for all), whereas patients diagnosed with venous thromboembolism had elevated levels of CD62E+, CD54+ and CD142+ CECs (*p* < 0.001 for all) in opposition to controls. In the VTE subgroup, associations of the amount of CECs and CD62E+ with the number of leukocytes (r = +0.515, *p* = 0.041; r = +0.605, *p* = 0.013), of CD142+ CECs with the thrombosis count (r = +0.568, *p* = 0.022) and of antithrombin levels with CD54+ CECs (r = +0.558, *p* = 0.025) were detected. In MPNs, irrespective of the *JAK2* mutational status, the researchers depicted associations of the amount of CECs, progenitor CECs and CD62E+ with the number of leukocytes (r = +0.738, *p* = 0.001; r = +0.846, *p* < 0.001; r = +0.610, *p* = 0.009), as well as a negative association of the number of thrombocytes and CD54+ CECs levels (r = −0.508, *p* = 0.037) [39].

Trelinski et al. (2010) investigated the levels of CECs in ET versus healthy subjects and registered elevated total, activated, resting, progenitor, CD46+ and apoptotic CECs concentrations (*p* = 0.001 for all) in ET. The results remained statistically significant irrespective of the treatment employed, i.e., untreated, treated with anagrelide or with hydroxyurea, and were not influenced by the presence of risk factors for thrombotic events, including *JAK2* gene mutations. Several angiogenesis-related molecules (vascular endothelial growth factor and soluble vascular endothelial growth factor receptor 1, but not soluble vascular endothelial growth factor receptor 2, angiopoietin-1 or angiopoietin-2) were detected in elevated levels in ET subjects, whereas controls had higher placenta growth factor levels [40].

The same group of Trelinski et al. (2010) analyzed the quantity of CECs in PV and ET. Similarly, total, activated, progenitor and apoptotic CECs were higher in MPN patients versus controls (*p* < 0.001 for all), irrespective whether the patients were diagnosed with PV or ET. However, resting CECs were only higher in ET versus controls (*p* < 0.001) and in ET versus PV patients (*p* < 0.05). In addition, apoptotic CECs were also elevated in ET versus PV (*p* < 0.05) and in PV subjects with >8700 leukocytes/mmc versus <8700 leukocytes/mmc (*p* = 0.04) [41].

The main findings of this systematic review are summarized in Table 1.

## 4. Discussion

In this systematic review, we conducted a comprehensive analysis of the literature regarding the potential role of liquid biopsy and the utility of cfDNA, EVs, MPs and CECs in the diagnosis, prognosis and monitoring of MPNs. Notably, in all of the analyzed studies, peripheral blood was collected for sample processing and evaluation, with flow cytometry peaking as the most employed technique to assess biomarkers of MPNs.

Although in solid cancers and several lymphoma subtypes [8,9,10,11], cfDNA was primarily detected by liquid biopsy, we retrieved only one study [15] employing cfDNA in MPNs. Based on the findings of that research, we may hypothesize that cfDNA could be used to discriminate between MPNs and healthy subjects and between MPNs subtypes, namely, to differentiate PMF from PV and ET [15]. In particular, we might be able to employ cfDNA to differentiate between MPNs and secondary causes of erythrocytosis, thrombocytosis and myelofibrosis, particularly when conventional techniques have failed to provide satisfactory results. Moreover, as thrombosis remains a challenge in the management of MPNs [5,6,7], the early identification of the patients at risk for such complications, before the development of clinical symptoms, based on cfDNA levels might be an interesting option, as Garcia-Gisbert et al. (2021) pointed out that MPNs subjects who experienced thrombotic complications had higher cfDNA levels. MPNs subjects are at high risk of venous, but also arterial, thrombosis, with thrombotic events emerging, in many instances, as the inaugural event in MPNs diagnosis and guiding the selection of risk-adapted therapy [42,43,44,45].

The study of EVs revealed increased levels of MVs, PaCs and PLT-EVs and decreased MK-EVs in samples collected from MPN subjects, and in particular in patients with PMF [16,17,18,19,20,21,22]. PLT-EVs have been shown to contribute to the development of cardiovascular disease and cancer, in addition to their contributory role in the occurrence of thrombosis and can influence hematopoiesis during inflammatory states via bone marrow infiltration [46,47,48]. Moreover, their involvement in the pathogenesis of other hematological malignancies, e.g., multiple myeloma, acute myeloid leukemia, chronic myeloid leukemia or chronic lymphocytic leukemia, has been demonstrated. In particular, in the aforementioned disorders, EVs have displayed a procoagulant activity and have been shown to express TF [49]. Thrombosis in MPNs is a complex event and requires the crosstalk of WBCs, PLTs, RBCs, inflammation and endothelial cells and the discovery of early messengers of vascular events in these blood cancers is warranted [50].

MPs and CECs constituted the vast majority of the biomarkers explored in our systematic review [23,24,25,26,27,28,29,30,31,32,33,34,35,36,37,38,39,40,41], probably because their detection from peripheral blood samples via flow cytometry is accessible and less time-consuming in comparison with other laboratory techniques, with flow cytometry playing a key role in the diagnosis of several hematological malignancies and in minimal residual disease monitoring [51]. The involvement of PLTs, PLTMVs and PMPs in malignancies has been well-documented in the literature, with these elements working together with inflammatory and neoplastic cells to enhance disease progression and favor the development of thrombotic complications [52]. Moreover, PMPs seem to confer protection against the action of chemotherapy, particularly in acute myeloid leukemia, as reported by Cacic et al. (2021) [53]. In addition, in malignant diseases, there is a link between TF+MPs and the recurrence of VTE [54]. Furthermore, RMPs and EMPs have been regarded as putative agents in the development of coronary heart disease and vascular events, e.g., acute myocardial infarction, whereas GMPs and other WBCs-derived MPs interact with immune cells and enhance the production of pro-inflammatory cytokines, participating in the pathogenesis and evolution of cardiovascular and allergic disorders, infections, pre-eclampsia or cancer [55,56]. Overall, we may hypothesize that MPs and CECs can be employed as diagnostic tools in MPNs, to differentiate between MPNs subtypes and secondary causes of polycythemia, thrombocytosis or myelofibrosis, and also to predict the occurrence of vascular events.

As the management of solid and blood cancers evolves towards less invasive techniques, in the near future, liquid biopsy and liquid biopsy-based biomarkers might emerge as strategic tools in the diagnosis, evaluation and management of MPNs. It is too early to call whether it can replace bone marrow biopsy, but this hypothesis should be explored in future research. It would be helpful to explore the utility of liquid biopsy and the aforementioned peripheral blood biomarkers to resolve some unmet needs in the diagnosis and management of MPNs. For example, early-stage (prefibrotic) PMF and ET can be distinguished only by performing a bone marrow biopsy which is an investigation not easily tolerated by patients and definitely unpleasant in comparison to drawing blood samples from peripheral veins. However, ET is associated with better overall survival and risk of transformation to acute myeloid leukemia versus prefibrotic PMF and thus the distinction between these two entities is mandatory [1]. In addition, as thrombosis has emerged as one of the main contributors to the increased morbidity and mortality of subjects diagnosed with MPNs, identifying new biomarkers with the potential to predict or at least alarm for the risk of thrombotic events would be of paramount importance. For example, median survival decreases with nearly 30 months and the risk of death increases (adjusted HR = 1.60, 95% CI = 1.01–2.51 for MPNs patients diagnosed with pulmonary embolism) in MPNs subjects who experience VTE versus those who did not [57]. Moreover, in patients whose complete blood count displays leukocytosis, the relative risk of thrombosis is elevated in ET subjects and in ET and PV subjects with a positive history for arterial thrombosis [58]. Our systematic review identified that MPNs patients who experience thrombosis exhibit elevated cfDNA (both at diagnosis and during follow-up), CECs, MPs (>4600 MPs/µL), TF+MPs (>87.7 TF + MPs/µL), PCA of MPs and reduced PMPs [15,26,30,31,36]. An interesting research hypothesis for future studies would be to combine the results of these assessments together with the presence of leukocytosis into the development of a thrombosis prediction score based on disease biomarkers. The evaluation of cfDNA is cheaper and less time-consuming versus granulocyte DNA studies which represents a major advantage of this novel technique [15]. Moreover, flow cytometry was the most employed method of biomarker evaluation in our systematic review. Flow cytometry is already widely used in the diagnosis of leukemia in hematology laboratories [51]. A main advantage of employing it is that there are already well-skilled professionals that perform flow cytometry on daily basis and thus training them to detect MPs, CECs or EVs would not imply extensive costs, although further research is needed to confirm this. One of the pros of detecting peripheral blood-based biomarkers is that their quantification, particularly by flow cytometry, is faster versus the pathology examination of bone marrow biopsy specimens. However, the number of published research on the topic of cfDNA, EVs, MPs and CECs in MPNs is limited and we are still uncertain to which extent we can rely on these parameters which is a drawback regarding the applications of liquid biopsy and liquid biopsy-based biomarkers in these blood cancers. In addition, as most of the evaluated studies measured the aforementioned biomarkers during follow-up, future research endeavors should be focused to evaluate these molecules at diagnosis or from samples collected at diagnosis. 

Our systematic review has several strengths, but also some limitations. Firstly, this is the first systematic review to explore the potential applications of liquid biopsy and liquid biopsy-based biomarkers in MPNs. Due to its design as a systematic review, the clear research question and the methodology allowed us to include a great deal of evidence. Moreover, we employed clear inclusion and exclusion criteria and we searched multiple databases to provide a comprehensive overview of the topic without an over/misinterpretation of the results. However, some shortcomings of our study were that it included observational studies and that a meta-analysis was not tempted due to the heterogeneity of the types of biomarkers and methods of detection employed. Another limitation is that a third of the examined papers were published in or earlier than 2011, i.e., nearly a decade or more ago. Thus, further research is needed to confirm our findings.

## 5. Conclusions

Liquid biopsy and liquid biopsy-based biomarkers could be used in MPNs for diagnostic and prognostic purposes. Future research is needed to clarify whether this technique can be employed to differentiate between MPN subtypes and secondary causes of erythrocytosis, thrombocytosis and myelofibrosis, as well as to predict the development of thrombosis.

## Figures and Tables

**Table 1:** Main results of the systematic review of the studies investigating the potential applications of liquid biopsy and liquid biopsy-based biomarkers in MPNs.

Author and Year	Study Location	Study Design	MPN	Patients No., Time Point	Parameters Assessed	Methods of Evaluation	Main Results
Garcia-Gisbert et al. (2020) [15]	Spain	Cross-sectional	PV, ET, PMF	107 diagnosis follow-up	cfDNA	DNA isolation	↑ cfDNA ↑ cfDNA, cfDNA/WBCs in PMF vs. PV, ET ↑ cfDNA in MPNs with thrombosis at **diagnosis/during follow-up** ↑ VAF *JAK2*, *MPL*, *SRSF2* in cfDNA vs. granulocyte DNA
Barone et al. (2019) [16]	Italy	Cross-sectional	ET, PMF, SMF	81 follow-up	PLTMVs, MKMVs, EMVs, MMVs,	Flow cytometry	↓ MKMVs in *JAK2*(+)/*CALR*(+)/TN MF ↓ MKMVs, ↑ PLTMVs in MF & ET ↑ PLTMVs in ET *vs.* MF ↑ PLTMVs in *JAK2*(+)/*CALR*(+) MF *vs.* TN MPNs or controls ↓ PLTMVs in TN vs. *JAK2*(+)/*CALR*(+) MF ↓ MKMVs, ↑ PLTMVs in *JAK2*(+)/*CALR*(+) ET vs. controls & TN-ET ↑ PLTMVs, ↓ MKMVs in high/intermediate 2 vs. low/intermediate-1 risk MF & controls MKMVs in MF: (+) correlation wit PLTs, (−) with IL-6 PLTMVs in MF: (−) correlation with splenomegaly degree, (+) with P-selectin, thrombopoietin ↓ PLTMVs, ↑MKMVs in RUX spleen-responders in MF at baseline MKMVs <19.95% = spleen non-responders RUX ↓PLTMVs, ↑MKMVs in spleen-responders at 6 months ↑EMVs, MMVs in MF RUX ↓ EMVs in spleen-responders
Barone et al. (2020) [17]	Italy	Experimental	MF	30 diagnosis	MEVs-CK	Flow cytometry	RUX ↑ IL-1β, IL-6, TNF-α in LPS-stimulated MF monocytes
Poisson et al. (2020) [18]	France	Experimental	JAK2V617F(+) MPNs	7 follow-up	Plasma MVs	Flow cytometry	MVs from *JAK2* V617F-positive MPNs ↑phenylephrine-induced contraction in mice aorta
Pecci et al. (2015) [19]	Italy	Cross-sectional	PV, ET, PMF	5 follow-up	PaCS, proteasome levels	EM+IGA, WB, ELISA	↑ PaCS in PLTs, granulocytes ↑ proteasome levels in PLTs and granulocytes extracts ↑ proteasome levels in plasma
Caivano et al. (2015) [20]	Italy	Cross-sectional	PMF	5 follow-up	MVs	Flow cytometry	↑ small-diameter MVs
Fel et al. (2019) [21]	Germany	Case-control	PV	9 follow-up	EVs	Liquid chromatography, tandem mass spectrometry	↑ CD42d+, CD71+, CD62L+ cells in PV ↑ APs, ↑ inflammatory/immune/angiogenic/procoagulant markers ↑ 13x transferrin receptor protein 1 ↑ 11.2x heparanase ↑ 5–6x plasminogen activator inhibitor 1, histone H4 and H2B, angiogenin ↑ 4–5x matrix metalloproteinase-9, neurogenic locus notch homolog protein 3, lysozyme C, histone H3, L-selectin, lactotransferrin, solute carrier family 2 ↑ 3–4x coagulation factor XI, myeloperoxidase, C-reactive protein, vinculin, platelet multimerin-1
Forte et al. (2011) [22]	Italy	Cross-sectional	PMF	29 follow-up	EVs	Flow cytometry	↓ MK-EVs in *JAK2* V617F (+) & TN-MF ↓ PLT-EVs in TN-MF, controls vs. *JAK2* V617F (+) MF ↑ in vitro survival, ↑ miR-361-5p of TN-MF EVs miR-34a-5p, miR-222-3p, miR-361-5p upregulated in *JAK2* V617F (+) & TN-MF miR-127-3p upregulated in *JAK2* V617F (+) MF (+) miR-34a-5p, (−) miR-212-3p & *JAK2* V617F VAF association
Zhang et al. (2017) [23]	China	Cross-sectional	PV, ET, PMF	92 follow-up	PMPs, EMPs, RMPs, TF+MPs	Flow cytometry	↑ RMPs, ↑ PMPs, ↑ EMPs, ↑ TF+MPs PMF vs. PV: ↑ RMPs, ↑ PMPs, ↑ EMPs, ↑ TF+MPs ET vs. PV: ↑ EMPs
Villmow et al. (2003) [24]	Germany	Cross-sectional	PV, ET, PMF	37 follow-up	PMPs, PMAs, APs, PNCs, PMCs	Flow cytometry	↑ APs ↑ PMPs in PV, ET, MF vs. CML, controls ↑ PNCs, ↑PMCs in ET, PV vs. MF, CML, controls
Trappenburg et al. (2009) [25]	Italy	Cross-sectional	ET	21 follow-up	PMPs, EMPs, GMPs, MMPs, TF+MPs	Flow cytometry	↑ MPs, ↑ CD61(+) PMPs, ↓ CD63(+) PMPs, ↑ vWF, ↑ TF+MPs ↑ EMPs, i.e., CD62E(+), CD144(+) ↑ GMPs, i.e., CD66b(+) and CD66acde(+) ↑ MMPs, i.e., CD14(+) ↑ CD62E(+)/CD41(+) EMPs in ET with ↑ CV risk
Taniguchi et al. (2017) [26]	Japan	Cross-sectional	PV, ET, PMF, SMF	59 follow-up	PMPs, EMPs, TF+MPs	Flow cytometry	cytoreduction ↓ procoagulant, annexin V(+) MPs, ↓ TF+MPs anticoagulation ↓ MPs in MPNs 70% of annexin V(+) MPs = PMPs, i.e., CD41a(+) 30% of annexin V(+) MPs = EMPs, i.e., CD146(+), or CD45(+), i.e., leukocyte-derived history of thrombosis +/− no cytoreduction =↑ TF+MPs >84.7 TF+MPs/µL, documented CV risk = predictors of thrombosis in MPNs
Tan et al. (2013) [27]	China	Cross-sectional	PV	23 follow-up	PMPs, GMPs, EMPs, RMPs, TF+MPs	Flow cytometry	↑MPs, ↑ PMPs, ↑ RMPs, ↑ GMPs, ↑ EMPs in PV vs. SP or controls ↑ PS(+) PLTs, RBCs in PV vs. SP or controls ↓ clotting time, ↑thrombin and FXase generation in PV HU ↓ MPs, ↑ PMPs, ↑ RMPs, PS(+) PLTs/RBCs in PV
Piccin et al. (2017) [28]	Italy	Cross-sectional	JAK2V617F(+) ET	66 follow-up	PMPs, EMPs, RMPs, TF+MPs	Flow cytometry	↓ MPs, ↓ PMPs, ↑ NO, ↑ ADM in ET on ASA, HU+ASA ↑ EMPs, ↑ RMPs in untreated ET ↑ EMPs in ET vs. controls ↓ EMPs, ↓ ED-1 in ET on ASA+ANA
Ahadon et al. (2018) [29]	Malaysia	Case-control	PV	15 diagnosis	PMPs, EMPs	Flow cytometry	↑ PMPs
Aswad et al. (2019) [30]	Czech Republic	Cross-sectional	PV, ET, PMF	179 diagnosis follow-up	PMPs, RMPs	Flow cytometry, functional coagulation assays	↑ PMPs, RMPs ↑ PMPs in PV, ET vs. PMF ↑ procoagulant activity of MPs association of PMPs procoagulant activity and PMPs levels ↓ PMPs in MPNs with (+) history of thrombosis ↑ PMPs in *JAK2* V617F (+) MPNs PMPs correlated with Hb, Ht, RBCs, PLTs, WBCs
Charpentier et al. (2016) [31]	France	Cross-sectional	ET	74 diagnosis	PMPs, RMPs, MMPs, GMPs, EMPs	Flow cytometry	↑ total MPs, RMPs, PMPs in *JAK2* V617F (+) vs. *CALR*(+)/TN-ET (+) associations of MPs with thrombin generation, phospholipid-dependent procoagulant activity ↑ procoagulant activity in *JAK2* V617F (+) vs. *CALR*(+)/TN-ET ↑ MPs in high vs. intermediate/low thrombotic risk ET >4600 MPs/µL = high-risk of thrombosis in ET
Marchetti et al. (2014) [32]	Italy	Cross-sectional	ET	73 follow-up	MPs	Flow cytometry	↑ ETP, ↑ peak of thrombin, ↓ lag-time, ↓ time to peak, ↓ clotting times ↑ ETP, ↑ peak of thrombin, ↓ lag-time, ↓time to peak in *JAK2* V617F (+) vs. (−) ↓ clotting times in *JAK2* V617F (+) vs. (−) *JAK2* V617F predicts shortened clotting times (+) association of PCA and lag-time, time to peak (−) association of PCA and peak of thrombin, ETP removal of MPs ↓ EDT, ↑ clotting times in ET, controls ↑ TF, ↑ FVIIa/AT in *JAK2* V617F (+) ET
Moles-Moreau et al. (2009) [33]	France	Cross-sectional	ET	37 diagnosis	PMPs	Flow cytometry	↑ PMPs, PMPs/PLTs, ↑ CD36+ cells ↑ PMPs/PLTs ratio, ↑ CD36+ cells in ET vs. RT ↑ PMPs, PMPs/PLTs ratio in RT vs. controls
Connor et al. (2013) [34]	Australia	Cross-sectional	ET	10 follow-up	PMPs	Flow cytometry	↑PMPs
Duchemin et al. (2010) [35]	France	Cross-sectional	PV, ET	44 follow-up	MPs	Functional assays	↑ CPA, ↓ ETP in MPNs pre-/post-filtration of MPs ↑ CPA in *JAK2* V617F (+), homozygous genotype ↑ TM-resistance, ↓free protein S (+) association of CPA and neutrophils, RBCs, PLTs (−) association of ETP and *JAK2* V617F allele burden ↑ CPA, ↓ ETP in *JAK2* V617F homozygous genotype MPNs post-filtration of MPs
Kissova et al. (2015) [36]	Czech Republic	Cross-sectional	PV, ET, PMF	126 follow-up	MPs	Flow cytometry	↑ MPs ↑ PCA of MPs in PV vs. ET/ PMF ↑ PCA of MPs in *JAK2* V617F (+) vs. (−) MPNs (+) association of PCA of MPs with Hb, Ht in PV association of PCA with PLTs ↑ PCA of MPs in MPNs with venous thrombosis history cytoreduction ↓PCA of MPs
Alonci et al. (2008) [37]	Italy	Cross-sectional	PV, ET, PMF	40 follow-up	CECs	Flow cytometry	↑ CD34+ CECs, ↑ CD34+ CD133- VEGFR2+ CECs ↑ CD34+ CECs in PMF vs. ET, PV ↑ CD34+ CD133+ VEGFR2+ CECs in PMF, PV vs. controls, ET ↑ CD34+ CD133- VEGFR2+ CECs in PMF vs. PV
Belotti et al. (2011) [38]	Italy	Cross-sectional	ET	39 follow-up	CECs	Flow cytometry	↑ CD146+ CD45-CECs, soluble E-selectin
Torres et al. (2013) [39]	Portugal	Cross-sectional	PV, ET	17 follow-up	CECs	Flow cytometry	↑ MPNs, VTE vs. controls ↓ progenitor CECs in VTE vs. MPNs, controls ↑ CD62E+ CECs in MPNs vs. controls ↑ CD62E+, CD54+, CD142+ CECs in VTE vs. controls (+) associations of WBCs with total CECs, progenitor CECs, CD62E+CECs (−) associations of PLTs with CD54+CECs
Trelinski et al. (2010) [40]	Poland	Cross-sectional	ET	65 diagnosis follow-up	CECs	Flow cytometry	↑total, activated, resting, progenitor, CD46+, apoptotic CECs, VEGF, soluble VEFGR 1 ↓ placenta growth factor
Trelinski et al. (2010) [41]	Poland	Cross-sectional	PV, ET	46 follow-up	CECs	Flow cytometry	↑ total, activated, progenitor, apoptotic CECs ↑ resting CECs in ET versus PV, controls ↑ apoptotic CECs in ET versus PV ↑apoptotic CECs in PV with >8700 vs.<8700 WBCs

ADM, adrenomedullin. ANA, anagrelide. APs, activated platelets. ASA, acetylsalycilic acid. *CALR*, calreticulin. CD, cluster of differentiation. CEC, circulating endothelial cells. cfDNA, cell-free DNA. CK, cytokine(s). CML, chronic myeloid leukemia. CPA, circulating procoagulant activity of plasma. CV risk, cardiovascular risk. ED-1, endothelin-1. ELISA, enzyme-linked immunoassay. EM+IGA, electron microscopy and immunogold analysis. EMPs, endothelial MPs. EMVs, endothelial MVs. ET, essential thrombocythemia. ETP, endogeneous thrombin potential. GMPs, granulocyte-derived MPs. Hb, hemoglobin. Ht, hematocrit. IL-6, interleukin-6. LDH, lactate dehydrogenase. MEVs-CK, monocyte-derived extracellular vesicles. MF, myelofibrosis (unspecified whether primary or secondary). miR, microRNA. MKMVs, megakaryocyte MVs. MMPs, monocyte-derived MPs. MMVs, monocyte MVs. MP, microparticles. MPN, myeloproliferative neoplasms. MPNu, MPN unclassfiable. MVs, microvesicles. PaCS, particulate cytoplasmic structures. PCA, procoagulant activity. PLTs, platelets. PLTMVs, platelet MVs. PMF, primary mielofibrosis. PMAs, platelet microaggregates. PMPs, platelet-derived MPs. PMCs, platelet-monocyte conjugates. PNCs, platelet-neutrophil conjugates. PS, phosphatidylserine. PV, polycythemia vera. RBCs, red blood cells. RMPs, red blood cell MPs. RT, reactive thrombocytosis. RUX, ruxolitinib. SMF, secondary MF. SP, secondary polycythemia. TFMPs, tissue factor-positive MPs. TN, triple-negative. VAF, variant allele frequency. VEFGR2, vascular endothelial growth factor receptor 2. VTE, venous thromboembolism. vs, versus. WB, Western Blot. WBCs, white blood cell count (leukocytes). ↑, increased. ↓, decreased. (+), positive. (−), negative.

## Supplementary Materials

The following are available online at https://www.mdpi.com/article/10.3390/life11070677/s1, Table S1: Main results of the systematic review of the studies investigating the potential applications of liquid biopsy and liquid biopsy-based biomarkers in MPNs, Table S2: Methodological quality assessment of the included studies, Table S3: Risk of bias assessment of the included studies.

## Abbreviations

ADM	adrenomedullin
ANA	anagrelide
APs	activated platelets
ASA	acetylsalycilic acid
CALR	calreticulin
CD	cluster of differentiation
CEC	circulating endothelial cells
cfDNA	cell-free DNA
CK	cytokine(s)
CML	chronic myeloid leukemia
CPA	circulating procoagulant activity of plasma
CV risk	cardiovascular risk
ED-1	endothelin-1
ELISA	enzyme-linked immunoassay
EM+IGA	electron microscopy and immunogold analysis
EMPs	endothelial MPs
EMVs	endothelial MVs
ET	essential thrombocythemia
ETP	endogeneous thrombin potential
GMPs	granulocyte-derived MPs
Hb	hemoglobin
Ht	hematocrit
IL-6	interleukin-6
LDH	lactate dehydrogenase
MEVs-CK	monocyte-derived extracellular vesicles
MF	myelofibrosis (unspecified whether primary or secondary)
miR	microRNA
MKMVs	megakaryocyte MVs
MMPs	monocyte-derived MPs
MMVs	monocyte MVs
MP	microparticles
MPN	myeloproliferative neoplasms
MPNu	MPN unclassifiable
MVs	microvesicles
PaCS	particulate cytoplasmic structures
PCA	procoagulant activity
PLTs	platelets
PLTMVs	platelet MVs
PMF	primary myelofibrosis
PMAs	platelet microaggregates
PMPs	platelet-derived MPs
PMCs	platelet-monocyte conjugates
PNCs	platelet-neutrophil conjugates
PS	phosphatidylserine
PV	polycythemia vera
RBCs	red blood cells
RMPs	red blood cell MPs
RT	reactive thrombocytosis
RUX	ruxolitinib
SMF	secondary MF
SP	secondary polycythemia
TFMPs	tissue factor-positive MPs
TN	triple-negative
VAF	variant allele frequency
VEFGR2	vascular endothelial growth factor receptor 2
VTE	venous thromboembolism
vs	versus
WB	Western Blot
WBCs	white blood cell count (leukocytes)
↑	increased
↓	decreased
(+)	positive
(−)	negative
