# Supplementary material for: Liquid Biopsy and Potential Liquid Biopsy-Based Biomarkers in Philadelphia-Negative Classical Myeloproliferative Neoplasms: A Systematic Review
Source: Life (Basel). 2021 Jul 10;11(7):677. doi: 10.3390/life11070677 (PMC8304270; doi:10.3390/life11070677)
Supplement: Supplementary file 1 [file life-11-00677-s001.zip › life-1271841-SI.pdf]

life MDPI

Supplementary files

Table S1. Main results of the systematic review of the studies investigating the potential applications of liquid biopsy and liquid biopsy-based biomarkers in MPNs.

Author and Year	Study location	Study design	MPN	Number of patients	Parameters assessed	Methods of evaluation	Main Results
Garcia-Gisbert et al. (2020) [15]	Spain	Cross-sectional	PV, ET, PMF, MPNu	107	cfDNA	DNA isolation (peripheral venous blood)	↑cfDNA in PV, ET, PMF vs controls<br>↑cfDNA, cfDNA/WBCs in PMF vs PV, ET<br>(+) association cfDNA-WBCs, cfDNA-LDH<br>↑cfDNA in MPNs with thrombosis at diagnosis/during follow-up<br>↑VAF of mutations in cfDNA vs granulocyte DNA<br>(+) association of cfDNA – granulocyte DNA, number of mutations/patient<br>↑VAF *JAK2*, *MPL*, *SRSF2* in cfDNA *vs* granulocyte DNA
Caivano et al. (2015) [20]	Italy	Cross-sectional	PMF	5	MVs	Flow-cytometry (peripheral venous blood)	↑small-diameter (0-0.3 μm) MVs in PMF *vs* controls
Poisson et al. (2020) [18]	France	Experimental	JAK2V617F(+) MPNs	7	Plasma MVs	Flow-cytometry (platelet poor plasma)	MVs from *JAK2* V617F-positive MPNs ↑phenylephrine-induced contraction in mice aorta
Barone et al. (2020) [17]	Italy	Experimental	MF	30	MEVs-CK	Flow-cytometry (peripheral venous blood)	IL-1β, IL-6, IL-10, TNF-α similar on MEVs in MF *vs* controls<br>LPS stimulation↑ IL-1β, IL-6, TNF-α production only in controls<br>RUX ↑ IL-1β, IL-6, TNF-α in LPS-stimulated MF monocytes
Pecci et al. (2015) [19]	Italy	Cross-sectional	PV, ET, PMF	5	PaCS, proteasome levels	EM+IGA (peripheral blood), WB (protein extracts), ELISA (plasma)	↑ PaCS in PLTs, granulocytes<br>↑proteasome levels in PLTs and granulocytes extracts of MPN subjects<br>↑proteasome levels in the plasma of MPN patients
Forte et al. (2011) [22]	Italy	Cross-sectional	PMF	29	EVs	Flow-cytometry (platelet-poor plasma)	↓MK-EVs in *JAK2* V617F (+) & TN-MF vs controls<br>↓PLT-EVs in TN-MF, controls vs *JAK2* V617F (+) MF<br>↑ in vitro survival, ↑ miR-361-5p of TN-MF EVs<br>miR-34a-5p, miR-222-3p, miR-361-5p upregulated in *JAK2* V617F (+) & TN-MF<br>miR-127-3p upregulated in *JAK2* V617F (+) MF<br>(+) miR-34a-5p & *JAK2* V617F VAF association<br>(-) miR-212-3p & *JAK2* V617F VAF association

Fel et al. (2019) [21]	Germany	Case-control	PV	9	EVs	Liquid chromatography, tandem mass spectrometry	↑CD42d+, CD71+, CD62L+ cells in PV<br>↑APs, ↑inflammatory/immune/angiogenic/procoagulant markers<br>↑13x transferrin receptor protein 1<br>↑11.2x heparanase<br>↑5-6x plasminogen activator inhibitor 1, histone H4 and H2B, angiogenin<br>↑4-5x matrix metalloproteinase-9, neurogenic locus notch homolog protein 3, lysozyme C, histone H3, L-selectin, lactotransferrin, solute carrier family 2<br>↑3-4x coagulation factor XI, myeloperoxidase, C-reactive protein, vinculin, platelet multimerin-1
Barone et al. (2019) [16]	Italy	Cross-sectional	ET, PMF, SMF	81	PLTMVs, MKMVs, EMVs, MMVs,	Flow-cytometry (peripheral venous blood)	↓ MKMVs, ↑ PLTMVs in MF & ET *vs* controls<br>↑ PLTMVs in ET *vs* MF<br>↑ PLTMVs in *JAK2*(+)/*CALR*(+) MF *vs* TN MPNs or controls<br>↓ MKMVs in *JAK2*(+)/*CALR*(+)/TN MF *vs* controls<br>↓ PLTMVs in TN vs *JAK2*(+)/*CALR*(+) MF<br>↓ MKMVs, ↑ PLTMVs in *JAK2*(+)/*CALR*(+) ET *vs* controls & TN-ET<br>↑ PLTMVs, ↓ MKMVs in high/intermediate 2 *vs* low/intermediate-1 risk MF & controls<br>MKMVs in MF : (+) correlation wit PLTs, (-) with IL-6<br>PLTMVs in MF : (-) correlation with splenomegaly degree, (+) with P-selectin, thrombopoietin<br>↓ PLTMVs, ↑MKMVs in RUX spleen-responders in MF at baseline<br>MKMVs <19.95% = spleen non-responders<br>RUX ↓PLTMVs, ↑MKMVs in spleen-responders at 6 months<br>↑EMVs, MMVs in MF<br>RUX ↓ EMVs in spleen-responders
Duchemin et al. (2010) [35]	France	Cross-sectional	PV, ET	44	MPs	Functional assays (platelet-poor plasma)	↑CPA, ↓ETP in MPNs *vs* controls pre-/post-filtration of MPs<br>↑CPA in *JAK2* V617F (+), homozygous genotype<br>↑TM-resistance, ↓free protein S in MPNs *vs* controls<br>(+) association of CPA and neutrophils, RBCs, PLTs<br>(-) association of ETP and *JAK2* V617F allele burden<br>↑CPA, ↓ETP in *JAK2* V617F homozygous genotype MPNs post-filtration of MPs
Charpentier et al. (2016) [31]	France	Cross-sectional	ET	74	PMPs, RMPs, MMPs, GMPs, EMPs	Flow-cytometry (platelet-poor plasma)	↑ total MPs, RMPs, PMPs in *JAK2* V617F (+) vs *CALR*(+)/TN-ET<br>similar MMPs, GMPs, EMPs in all molecular subtypes of ET

							similar MPs (including subtypes) in *CALR*(+)/TN-ET<br>(+) associations of MPs with thrombin generation, phospholipid-dependent procoagulant activity<br>↑procoagulant activity in *JAK2* V617F (+) *vs* *CALR*(+)/TN-ET<br>↑MPs in high *vs* intermediate/low thrombotic risk ET<br>>4600 MPs/μL = high-risk of thrombosis in ET
Aswad et al. (2019) [30]	Czech Republic	Cross-sectional	PV, ET, PMF	179	PMPs, RMPs	Flow-cytometry, functional assays for coagulation (platelet-poor plasma)	↑PMPs, RMPs in MPNs *vs* controls<br>↑ PMPs in PV, ET *vs* PMF<br>similar RMPs in PV, ET, PMF<br>↑procoagulant activity of MPs in MPNs *vs* controls<br>association of PMPs procoagulant activity and PMPs levels<br>↓PMPs in MPNs with (+) history of thrombosis<br>↑ PMPs in *JAK2* V617F (+) MPNs<br>PMPs correlated with Hb, Ht, RBCs, PLTs, WBCs
Ahadon et al. (2018) [29]	Malaysia	Case-control	PV	15	PMPs, EMPs	Flow-cytometry (plasma)	↑PMPs in PV vs controls<br>EMPs similar in PV and controls
Piccin et al. (2017) [28]	Italy	Cross-sectional	JAK2V617F(+) ET	66	PMPs, EMPs, RMPs, TF+MPs	Flow-cytometry (platelet poor plasma)	↓MPs, ↓PMPs, ↑NO, ↑ADM in ET on ASA, HU+ASA<br>↑EMPs, ↑RMPs in untreated ET<br>↑EMPs in ET *vs* controls<br>↓EMPs, ↓ED-1 in ET on ASA+ANA<br>TF+MPs similar in all groups
Tan et al. (2013) [27]	China	Cross-sectional	PV	23	PMPs, GMPs, EMPs, RMPs, TF+MPs	Flow-cytometry (platelet poor plasma)	↑MPs, ↑PMPs, ↑RMPs, ↑GMPs, ↑EMPs in PV *vs* SP or controls<br>TF+MPs similar in PV, SP, controls<br>↑PS(+) PLTs, RBCs in PV *vs* SP or controls<br>↓clotting time, ↑thrombin and FXase generation in PV<br>HU ↓MPs, ↑PMPs, ↑RMPs, PS(+) PLTs/RBCs in PV
Taniguchi et al. (2017) [26]	Japan	Cross-sectional	PV, ET, PMF, SMF	59	PMPs, EMPs, TF+MPs	Flow-cytometry (platelet poor plasma)	cytoreduction ↓ procoagulant, annexin V(+) MPs, ↓TF+MPs<br>anticoagulation ↓ MPs in MPNs<br>70% of annexin V(+) MPs = PMPs, i.e., CD41a(+)<br>30% of annexin V(+) MPs = EMPs, i.e., CD146(+), or CD45(+), i.e., leukocyte-derived<br>history of thrombosis +/- no cytoreduction =↑TF+MPs<br>>84.7 TF+MPs/μL, documented CV risk = predictors of thrombosis in MPNs
Trappenburg et al. (2009) [25]	Italy	Cross-sectional	ET	21	PMPs, EMPs, GMPs,	Flow-cytometry (plasma)	↑MPs, ↑CD61(+) PMPs, ↓CD63(+) PMPs, ↑vWF, ↑TF+MPs in ET *vs* controls<br>↑EMPs, i.e., CD62E(+) and CD144(+) in ET *vs* controls

					MMPs, TF+MPs		↑GMPs, i.e., CD66b(+) and CD66acde(+) in ET *vs* controls<br>↑MMPs, i.e., CD14(+) in ET *vs* controls<br>↑CD62E(+)/CD41(+) EMPs in ET with ↑CV risk
Connor et al. (2013) [34]	Australia	Cross-sectional	ET	10	PMPs	Flow-cytometry (platelet-rich plasma)	↑PMPs in ET *vs* controls
Villmow et al. (2003) [24]	Germany	Cross-sectional	PV, ET, PMF	37	PMPs, PMAs, APs, PNCs, PMCs	Flow-cytometry (whole blood)	↑APs in MPNs *vs* controls<br>↑PMPs in PV, ET, MF *vs* CML, controls<br>PMAs similar in MPNs, controls<br>↑PNCs, ↑PMCs in ET, PV *vs* MF, CML, controls
Zhang et al. (2017) [23]	China	Cross-sectional	PV, ET, PMF	92	PMPs, EMPs, RMPs, TF+MPs	Flow-cytometry (peripheral venous blood)	MPNs *vs* controls: ↑RMPs, ↑PMPs, ↑EMPs, ↑TF+MPs<br>PMF *vs* PV: ↑RMPs, ↑PMPs, ↑EMPs, ↑TF+MPs<br>ET *vs* PV: ↑EMPs
Moles-Moreau et al. (2009) [33]	France	Cross-sectional	ET	37	PMPs	Flow-cytometry (whole blood)	↑PMPs, PMPs/PLTs ratio, ↑CD36+ cells in ET vs controls<br>↑PMPs/PLTs ratio, ↑CD36+ cells in ET vs RT<br>similar PMPs in ET vs RT<br>↑PMPs, PMPs/PLTs ratio in RT vs controls
Marchetti et al. (2014) [32]	Italy	Cross-sectional	ET	73	MPs	Flow-cytometry (platelet-poor plasma)	↑ETP, ↑peak of thrombin in ET *vs* controls<br>↓lag-time, ↓time to peak in ET *vs* controls<br>↑ETP, ↑peak of thrombin, ↓lag-time, ↓time to peak in *JAK2* V617F (+) *vs* (-) ET<br>↓clotting times in ET vs controls, *JAK2* V617F (+) vs (-) ET<br>*JAK2* V617F predicts shortened clotting times in ET<br>(+) association of PCA and lag-time, time to peak<br>(-) association of PCA and peak of thrombin, ETP<br>removal of MPs ↓EDT, ↑clotting times in ET, controls<br>↑TF in ET vs controls<br>↑FVIIa/AT in *JAK2* V617F (+) ET vs controls
Kissova et al. (2015) [36]	Czech Republic	Cross-sectional	PV, ET, PMF	126	MPs	Flow-cytometry (platelet-poor plasma)	↑MPs in MPNs vs controls<br>↑PCA of MPs in PV vs ET/ PMF<br>↑PCA of MPs in *JAK2* V617F (+) vs (-) MPNs<br>(+) association of PCA of MPs with Hb, Ht in PV<br>association of PCA with PLTs<br>↑PCA of MPs in MPNs with venous thrombosis history<br>cytoreduction↓PCA of MPs
Alonci et al. (2008) [37]	Italy	Cross-sectional	PV, ET, PMF	40	CECs	Flow-cytometry (peripheral venous blood)	↑CD34+ CECs in PMF, ET, PV vs controls<br>↑CD34+ CECs in PMF vs ET, PV<br>↑CD34+ CD133+ VEGFR2+ CECs in PMF, PV vs controls, ET

							↑CD34+ CD133- VEGFR2+ CECs in PMF, PV, ET vs controls<br>↑CD34+ CD133- VEGFR2+ CECs in PMF vs PV
Belotti et al. (2011) [38]	Italy	Cross-sectional	ET	39	CECs	Flow-cytometry (peripheral venous blood)	↑CD146+ CD45- CECs in ET vs controls<br>↑soluble E-selectin w in ET vs controls
Torres et al. (2013) [39]	Portugal	Cross-sectional	PV, ET	17	CECs	Flow-cytometry (peripheral venous blood)	↑MPNs, VTE vs controls<br>↓progenitor CECs in VTE vs MPNs, controls<br>↑CD62E+ CECs in MPNs vs controls<br>↑CD62E+, CD54+, CD142+ CECs in VTE vs controls<br>(+) associations of WBCs with total CECs, progenitor CECs, CD62E+ CECs<br>(-) associations of PLTs with CD54+ CECs
Trelinski et al. (2010) [40]	Poland	Cross-sectional	ET	65	CECs	Flow-cytometry (peripheral venous blood)	↑total, activated, resting, progenitor, CD46+, apoptotic CECs in ET vs controls<br>↑VEGF, soluble VEFGR 1 in ET vs controls<br>↓placenta growth factor in ET vs controls
Trelinski et al. (2010) [41]	Poland	Cross-sectional	PV, ET	46	CECs	Flow-cytometry (peripheral venous blood)	↑total, activated, progenitor, apoptotic CECs in PV, ET<br>↑resting CECs in ET versus PV, controls<br>↑apoptotic CECs in ET versus PV<br>↑apoptotic CECs in PV with >8700 vs<8700 WBCs

ADM, adrenomedullin. ANA, anagrelide. APs, activated platelets. ASA, acetylsalycilic acid. *CALR*, calreticulin. CD, cluster of differentiation. CEC, circulating endothelial cells. cfDNA, cell-free DNA. CK, cytokine(s). CML, chronic myeloid leukemia. CPA, circulating procoagulant activity of plasma. CV risk, cardiovascular risk. ED-1, endothelin-1. ELISA, enzyme-linked immunoassay. EM+IGA, electron microscopy and immunogold analysis. EMPs, endothelial MPs. EMVs, endothelial MVs. ET, essential thrombocythemia. ETP, endogeneous thrombin potential. GMPs, granulocyte-derived MPs. Hb, hemoglobin. Ht, hematocrit. IL-6, interleukin-6. LDH, lactate dehydrogenase. MEVs-CK, monocyte-derived extracellular vesicles. MF, myelofibrosis (unspecified whether primary or secondary). miR, microRNA. MKMVs, megakaryocyte MVs. MMPs, monocyte-derived MPs. MMVs, monocyte MVs. MP, microparticles. MPN, myeloproliferative neoplasms. MPNu, MPN unclassfiable. MVs, microvesicles. PaCS, particulate cytoplasmic structures. PCA, procoagulant activity. PLTs, platelets. PLTMVs, platelet MVs. PMF, primary mielofibrosis. PMAs, platelet microaggregates. PMPs, platelet-derived MPs. PMCs, platelet-monocyte conjugates. PNCs, platelet-neutrophil conjugates. PS, phosphatidylserine. PV, polycythemia vera. RBCs, red blood cells. RMPs, red blood cell MPs. RT, reactive thrombocytosis. RUX, ruxolitinib. SMF, secondary MF. SP, secondary polycythemia. TFMPs, tissue factor-positive MPs. TN, triple-negative. VAF, variant allele frequency. VEFGR2, vascular endothelial growth factor receptor 2. VTE, venous thromboembolism. *vs*, versus. WB, Western Blot. WBCs, white blood cell count (leukocytes).↑, increased. ↓, decreased.(+), positive. (-), negative.

Table S2. Methodological quality assessment of the included studies.

**Study**	**Study design**	**Criteria**												**Total score**
		**1**	**2**	**3**	**4**	**5**	**6**	**7**	**8**	**9**	**10**	**11**	**12**	
Garcia-Gisbert et al. (2020)	Cross-sectional	2	1	2	2	2	1	0	0	1	2	1	2	16
Caivano et al. (2015)	Cross-sectional	2	0	2	2	2	0	0	0	1	2	1	2	14
Poisson et al. (2020)	Experimental	2	0	2	2	1	0	0	0	2	2	2	2	15
Barone et al. (2020)	Experimental	2	0	2	2	2	2	2	0	2	2	2	2	20
Pecci et al. (2015)	Cross-sectional	2	0	2	2	2	0	0	0	2	2	2	2	16
Forte et al. (2011)	Cross-sectional	2	0	2	2	2	0	0	0	2	2	2	2	16
Fel et al. (2019)	Case-control	2	0	2	2	2	0	0	0	2	2	2	2	16
Barone et al. (2019)	Cross-sectional	2	0	2	2	2	2	2	0	2	2	2	2	20

Duchemin et al. (2010)	Cross-sectional	2	2	2	2	2	0	0	0	2	2	2	2	18
Charpentier et al. (2016)	Cross-sectional	2	2	2	2	2	0	0	0	-	-	-	-	10
Aswad et al. (2019)	Cross-sectional	2	0	2	2	2	0	0	0	1	2	1	2	14
Ahadon et al. (2018)	Case-control	2	0	2	2	2	0	0	0	1	2	1	2	14
Piccin et al. (2017)	Cross-sectional	2	0	2	2	2	2	0	0	0	2	2	2	16
Tan et al. (2013)	Cross-sectional	2	2	2	2	2	0	0	0	2	2	2	2	18
Taniguchi et al. (2017)	Cross-sectional	2	0	2	2	2	1	1	0	1	1	1	2	15
Trappenburg et al. (2009)	Cross-sectional	2	2	2	2	2	1	1	2	2	2	2	2	22
Connor et al. (2013)	Cross-sectional	2	0	2	2	2	0	0	0	2	2	2	2	16
Villmow et al. (2003)	Cross-sectional	2	0	2	2	2	0	0	0	2	2	1	2	15
Zhang et al. (2017)	Cross-sectional	2	0	2	2	2	0	0	0	2	2	2	2	16
Moles-Moreau et al. (2009)	Cross-sectional	2	0	2	2	2	0	0	0	2	2	2	2	16
Marchetti et al. (2014)	Cross-sectional	2	2	2	2	2	0	0	0	2	2	2	2	18
Kissova et al. (2015)	Cross-sectional	2	2	2	2	2	1	0	0	2	2	1	2	18
Alonci et al. (2008)	Cross-sectional	2	0	2	2	2	0	0	0	2	2	2	2	16
Belotti et al. (2011)	Cross-sectional	2	0	2	2	2	2	2	0	2	2	2	2	20
Torres et al. (2013)	Cross-sectional	2	0	2	2	2	0	0	0	2	2	2	2	16
Trelinski et al. (2010)	Cross-sectional	2	0	2	2	2	0	0	0	2	2	2	2	16
Trelinski et al. (2010)	Cross-sectional	2	0	2	2	2	0	0	0	2	2	2	2	16

The methodological quality of the studies was assessed using the methodological index for non-randomized observational studies (MINORS) [13] which evaluates on a 0 to 2 scale the following criteria: 1. A clearly stated aim; 2. Inclusion of consecutive patients; 3. Prospective collection of data; 4. Endpoints appropriate to the aim of the study; 5. Unbiased assessment of the study endpoint; 6. Follow-up period appropriate to the aim of the study; 7. Loss to follow up less than 5%; 8. Prospective calculation of the study size; Additional criteria in the case of comparative studies: 9. An adequate control group; 10. Contemporary groups; 11. Baseline equivalence of groups; 12. Adequate statistical analyses. The items are scored 0 (not reported), 1 (reported but inadequate) or 2 (reported and adequate). The global ideal score being 16 for non-comparative studies and 24 for comparative studies.

Table S3. Risk of bias assessment of the included studies.

Study (quantitative descriptive)	Study design	MMAT criteria fulfilled				Risk of bias score
		1	2	3	4	
Garcia-Gisbert et al. (2020)	Cross-sectional	N	Y	Y	Y	***
Caivano et al. (2015)	Cross-sectional	N	Y	N	Y	**
Poisson et al. (2020)	Experimental	N	Y	Y	Y	***
Barone et al. (2020)	Experimental	N	Y	Y	Y	***
Pecci et al. (2015)	Cross-sectional	N	Y	N	Y	**
Forte et al. (2011)	Cross-sectional	N	Y	Y	Y	***
Fel et al. (2019)	Case-control	N	Y	Y	Y	***
Barone et al. (2019)	Cross-sectional	N	Y	Y	Y	***
Duchemin et al. (2010)	Cross-sectional	Y	Y	Y	Y	****
Charpentier et al. (2016)	Cross-sectional	Y	Y	Y	Y	****
Aswad et al. (2019)	Cross-sectional	N	Y	Y	Y	***
Ahadon et al. (2018)	Case-control	N	Y	Y	Y	***
Piccin et al. (2017)	Cross-sectional	N	Y	Y	Y	***
Tan et al. (2013)	Cross-sectional	Y	Y	Y	Y	****
Taniguchi et al. (2017)	Cross-sectional	Y	Y	Y	Y	****
Trappenburg et al. (2009)	Cross-sectional	Y	Y	Y	Y	****
Connor et al. (2013)	Cross-sectional	N	Y	Y	Y	***
Villmow et al. (2003)	Cross-sectional	N	Y	Y	Y	***
Zhang et al. (2017)	Cross-sectional	N	Y	Y	Y	***
Moles-Moreau et al. (2009)	Cross-sectional	N	Y	Y	Y	***
Marchetti et al. (2014)	Cross-sectional	Y	Y	Y	Y	****
Kissova et al. (2015)	Cross-sectional	Y	Y	Y	Y	****
Alonci et al. (2008)	Cross-sectional	N	Y	Y	Y	***
Belotti et al. (2011)	Cross-sectional	N	Y	Y	Y	***
Torres et al. (2013)	Cross-sectional	N	Y	Y	Y	***
Trelinski et al. (2010)	Cross-sectional	N	Y	Y	Y	***
Trelinski et al. (2010)	Cross-sectional	N	Y	Y	Y	***

The risk of bias of the analyzed studies was evaluated using the Mixed Methods Appraisal Tool (MMAT) [14] based for quantitative nonrandomized studies on 4 criteria: 1. Are participants (organizations) recruited in a way that minimizes selection bias? 2. Are measurements appropriate (clear origin, or validity known, or standard instrument; and absence of contamination between groups when appropriate) regarding the exposure/intervention and outcomes? 3. In the groups being compared (exposed vs. nonexposed; with intervention vs. without; cases vs. controls), are the participants comparable, or do researchers take into account (control for) the difference between these groups? 4. Are there complete outcome data (80% or above), and, when applicable, an acceptable response rate (60% or above), or an acceptable follow-up rate for cohort studies (depending on the duration of follow-up)? Each ∗ indicates percentage (25%, 50%, 75%, or 100%) of criteria met where ∗ (25%) corresponds to a high risk of bias and ∗∗∗∗ (100%) corresponds to a low risk of bias. Y: yes; N: no.

Keywords and word combinations used for the systematic search

("myeloproliferative neoplasm*" OR "myeloproliferative syndrome*" OR "polycythemia vera" OR "polycythaemia vera" OR "essential thrombocytosis*" OR "essential thrombocyth*" OR "myelofibro*") AND ("liquid biopsy" OR "circulating tumor cell" OR "circulating tumor cluster" OR "exosome" OR "ctDNA" OR "circulating tumor DNA" OR "cell free DNA" OR "cfDNA" OR "liquid biopsies" OR "circulating tumor cells" OR "circulating tumor clusters" OR "exosomes" OR "vesicle" OR "vesicles" OR "vesicular" OR "exosomal" OR "microparticle*" OR "circulating endothelial cell*").
